# Your Path to Transplant: a randomized controlled trial of a tailored computer education intervention to increase living donor kidney transplant

**DOI:** 10.1186/1471-2369-15-166

**Published:** 2014-10-14

**Authors:** Amy D Waterman, Mark L Robbins, Andrea L Paiva, John D Peipert, Crystal S Kynard-Amerson, Christina J Goalby, LaShara A Davis, Jessica L Thein, Emily A Schenk, Kari A Baldwin, Stacy L Skelton, Nicole R Amoyal, Leslie A Brick

**Affiliations:** Division of Nephrology, David Geffen School of Medicine at the University of California, Los Angeles, 10940 Wilshire Blvd, Suite 1223, Los Angeles, CA 90024 USA; Division of General Medical Sciences, Washington University School of Medicine, 660 S. Euclid Ave., Campus Box 8005 St. Louis, MO 63110 USA; Cancer Prevention Research Center, University of Rhode Island, 130 Flagg Rd., Kingston, RI 02881 USA; Renal and Pancreas Transplant Division, Saint Barnabas Medical Center, Livingston, NJ 07039 USA; College of Nursing, University of Missouri St. Louis, One University Blvd., St. Louis, MO 63121 USA

**Keywords:** Kidney transplantation, Living donor, Racial disparities, African-Americans, Hispanics, Patient education, Health knowledge/attitudes, Transtheoretical model

## Abstract

**Background:**

Because of the deceased donor organ shortage, more kidney patients are considering whether to receive kidneys from family and friends, a process called living donor kidney transplantation (LDKT). Although Blacks and Hispanics are 3.4 and 1.5 times more likely, respectively, to develop end stage renal disease (ESRD) than Whites, they are less likely to receive LDKTs. To address this disparity, a new randomized controlled trial (RCT) will assess whether Black, Hispanic, and White transplant patients’ knowledge, readiness to pursue LDKT, and receipt of LDKTs can be increased when they participate in the *Your Path to Transplant* (YPT) computer-tailored intervention.

**Methods/Design:**

Nine hundred Black, Hispanic, and White ESRD patients presenting for transplant evaluation at University of California, Los Angeles Kidney and Pancreas Transplant Program (UCLA-KPTP) will be randomly assigned to one of two education conditions, YPT or Usual Care Control Education (UC). As they undergo transplant evaluation, patients in the YPT condition will receive individually-tailored telephonic coaching sessions, feedback reports, video and print transplant education resources, and assistance with reducing any known socioeconomic barriers to LDKT. Patients receiving UC will only receive transplant education provided by UCLA-KPTP. Changes in transplant knowledge, readiness, pros and cons, and self-efficacy to pursue LDKT will be assessed prior to presenting at the transplant center (baseline), during transplant evaluation, and 4- and 8-months post-baseline, while completion of transplant evaluation and receipt of LDKTs will be assessed at 18-months post-baseline. The RCT will determine, compared to UC, whether Black, Hispanic, and White patients receiving YPT increase in their readiness to pursue LDKT and transplant knowledge, and become more likely to complete transplant medical evaluation and pursue LDKT. It will also examine how known patient, family, and healthcare system barriers to LDKT act alone and in combination with YPT to affect patients’ transplant decision-making and behavior. Statistical analyses will be performed under an intent-to-treat approach.

**Discussion:**

At the conclusion of the study, we will have assessed the effectiveness of an innovative and cost-effective YPT intervention that could be utilized to tailor LDKT discussion and education based on the needs of individual patients of different races in many healthcare settings.

**Trial registration:**

ClinicalTrials.gov, number NCT02181114.

## Background

Nationwide, there are approximately 615,000 patients with end-stage renal disease (ESRD), or kidney failure, a condition that necessitates renal replacement therapy through either dialysis or kidney transplantation to sustain life. Transplantation has clear survival and quality-of-life benefits for patients [1, 2] and can reduce national health-care costs [3]. Living donor kidney transplant (LDKT) is the optimal form of transplantation since it can occur more quickly than deceased donor kidney transplant (DDKT) [4, 5] and results in better graft survival [2] and better quality-of-life [6]. Because of the deceased donor organ shortage, most patients are now considering whether to receive kidneys from family and friends through LDKT.

Due to higher rates of diabetes and hypertension, the two primary causes of kidney failure [7], Blacks and Hispanics are 3.4 and 1.5 times more likely, respectively, to develop ESRD than their White or non-Hispanic counterparts [2]. Despite this, research has shown that, compared to Whites, Blacks and Hispanics are less likely to complete transplant medical evaluation [8–10], be placed on the waiting list or have longer wait times [11, 12], or receive DDKTs and LDKTs [2, 10, 13, 14]. Specifically, at 2 years post-wait-listing, approximately 20% of both Blacks and Hispanics had received transplants while 30% of Whites had (OPTN data as of 09/19/2014). Therefore, while increasing the rates of LDKT could increase the quality-of-life and decrease the mortality of all ESRD patients, this approach could have its greatest impact on Black and Hispanic patients’ lives.

Research has indicated that all patients, regardless of race, face many barriers to getting a LDKT, including a lack of knowledge about the advantages of LDKT [15], confusion about how to find living donors [16], concerns about risking a living donor’s health [15, 17], and fears about the possibility of the transplanted kidney failing [18, 19]. However, there are additional barriers to LDKT for Black and Hispanic patients. A recent study suggests that Hispanic ESRD patients may have low levels of knowledge about transplant, particular concerns about living donors’ wellbeing, and logistical barriers in the case of undocumented immigrants [20]. With Blacks and Hispanics less likely to donate kidneys when they die than Whites, the availability of matching deceased donor kidneys for this racial group is lower [21, 22]. For racial/ethnic minorities, unsuccessful completion of transplant medical evaluation and lower receipt of transplants is also exacerbated by lower socioeconomic status, greater levels of occupational insecurity, and more transient healthcare coverage compared to Whites [23–25]. Mistrust of healthcare providers is more common among Blacks and Hispanics than Whites [26, 27], which may affect their trust in physicians’ recommendations for LDKT and cause suspicion of LDKT itself [26, 28, 29]. Finally, variation in the availability and quality of a support network for minorities affects transplantation rates [9, 18, 30, 31].

In a previous trial, our research team designed a set of *Explore Transplant* (ET) print and video education materials to improve patients’ transplant knowledge and maximize informed LDKT decision-making [32]. Compared to patients in dialysis centers who received ET with those in centers who did not, after one month, patients in ET dialysis centers were more knowledgeable of transplant, had greater perception of transplant’s benefits, and were more likely to be reading and talking to others about LDKT [33]. One to two years later, more patients in ET dialysis centers were reactivating or starting transplant medical evaluation [33].

Although the results of this educational intervention were promising, there is evidence that interventions that are individually-tailored to ESRD patients’ needs and preferences may be even more successful at reducing racial disparities in completion of evaluation and receipt of LDKTs [34, 35]. In this manuscript, we describe the protocol of a new randomized controlled trial (RCT) to test the efficacy of a computerized *Your Path to Transplant* (YPT) educational program on decreasing racial disparities in LDKT. The RCT will determine, compared to a usual care education control group (UC) from the transplant center, whether Black, Hispanic, and White patients receiving YPT increase in their readiness to pursue LDKT and transplant knowledge, and whether they become more likely to complete transplant medical evaluation and pursue LDKT. It will also examine whether YPT’s effectiveness in changing LDKT decision-making and behavior is different between Blacks, Hispanics, and Whites. Finally, it will examine how known patient, family, and healthcare system barriers to LDKT act alone and in combination with YPT to affect Black and Hispanic patients’ transplant decision-making and behavior.

## Methods and study design

### Theoretical foundation of YPT

Guided by the Transtheoretical Model of Behavioral Change (TTM) [36], one proven approach for health promotion is to individually-tailor education by the level of patients’ readiness to take certain health behaviors and other decision-making variables, like the patients’ self-efficacy [37–42]. The TTM is particularly well-suited for the development of computer-tailored interventions (CTIs) that can be easily disseminated to entire populations. By utilizing relevant theory, normative databases and empirically-based decision rules, CTIs provide tailoring of the most appropriate information to each participant to guide their change process. This approach allows for thousands of unique combinations of individual feedback messages to be provided based on TTM constructs [38].

CTI feedback provided to patients via coaches and printed reports addresses how a specific patient’s knowledge and readiness to take a specific behavior compares to a normative group of patients (normative feedback) or changes over time compared to their own previous data (ipsative feedback). Individually-tailored interventions provide recommendations to educators to guide health conversations optimally that emphasize what is most helpful to the patient, minimize resistance, and emphasize key demographic or socioeconomic factors, if needed. Research has shown that patients who receive education tailored to their level of readiness have double the chance of taking action toward behavior change in the following 6 months [43].

### Design and advantages of YPT

YPT was developed by Dr. Amy Waterman, creator of the *Explore Transplant* (ET) Patient Education Program [32, 33], and experts in TTM behavior change, Drs. Mark Robbins and Andrea Paiva. For several reasons, we anticipate that the YPT educational program may be more effective than usual care education provided by transplant centers (Table 1). A unique and innovative element of YPT is the TTM-based CTI using validated transplant decision-making measures [44, 45] that can be delivered in person, via the telephone, and on the computer. First, the computer-generated content within this program is individually-tailored to patients’ levels of readiness to pursue DDKT and LDKT. In addition to their level of readiness, behavioral guidance is based on TTM constructs (including decisional balance and self-efficacy), addresses their unique gaps in knowledge, and discusses information relevant to any personal challenges patients could be facing to derail them from transplantation. Since the YPT assessment can be given at different time points throughout evaluation, patients’ changes in knowledge, readiness, and other TTM constructs over time can be assessed and tailored feedback communicated by coaches. Second, the program is delivered over four time points in small increments, recommending patients take small, more manageable steps toward pursuing LDKT. This empowers patients to feel they are making meaningful progress toward LDKT and honors that many ESRD patients experience mental fatigue when too much educational information is provided at one sitting. Third, since the YPT program acknowledges that DDKT and LDKT are two separate treatment options and choices, it addresses patients’ individual fears and readiness for DDKT and LDKT separately. Finally, the YPT program provides community-based resources and coaching to help overcome socioeconomic and other practical barriers that may derail transplantation.

**Table 1:** **Advantages of Your Path to Transplant computer-tailored education**

Innovation	Implementation	Resource
Tailors education to individual patient needs	□ Tailored based on individual patients’ transplant knowledge and decision-making	TTM-based computer–tailored intervention that was designed to track a patient’s attitudinal shifts over time
	□ Provides information and recommends steps to pursue transplant in small doses at multiple time points	
	□ Tracks patients’ knowledge and attitudinal shifts over time, allowing coaches to acknowledge growth and change	
Provides education in multiple media/formats	□ Provides education in multiple formats to account for multiple learning styles, including video stories, provider-led coaching sessions, and individually-tailored reports	Cultural competency-trained coaches providing individualized coaching to the patient and disseminating educational pamphlets, factsheets and a DVD that highlights the experiences of kidney donors and recipients
	□ Includes an in-person educational session with each patient to develop greater relationship between the patients and the coaches	
	□ Provides a culturally competent and socioeconomically sensitive coach to help guide patients through the education and transplant process for deceased and living donor transplant separately	
Engages external resources to help patients pursue transplant	□ Includes educational resources to engage patients’ support networks and potential living donors to learn about transplant along with the patient	A guide for family and friends is available for those family members and close friends of patients who want to learn about kidney transplant
	□ Provides resources to help patients overcome socioeconomic barriers limiting their access to transplant	
		Patients are given a 43 page community resource guide that contains information ranging from dental services to low cost transportation in the LA County area

To ensure that the individually-tailored feedback is medically accurate and culturally competent, we recruited an Advisory Board of key content and medical experts including transplant coordinators, nephrologists, kidney recipients, and researchers with expertise in developing culturally sensitive interventions that address the needs of low health literacy groups. The Advisory Board has advised in the development of, and approved of, all components of our educational intervention.

### YPT educational components

#### YPT tailored feedback

**Figure 1:**
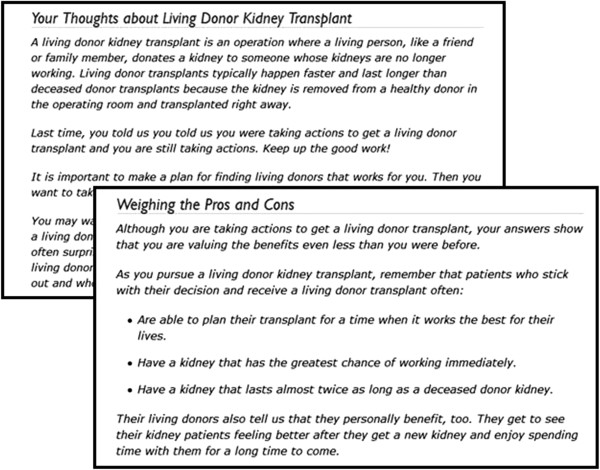
**Example feedback reports.**

The YPT individually-tailored feedback report includes sections about the patient’s knowledge about transplant (DDKT and LDKT), readiness to pursue transplant, perceived pros/cons to transplant, confidence in pursuing transplant, and potential socioeconomic barriers to transplant (Figure 1 and Table 2). As prompted by bulleted coaching points generated by the YPT CTI, the coach will discuss gaps in a patient’s specific transplant knowledge based on what questions they missed and will recommend small next steps to take (e.g., “Ask another person to tell others about your need for a living donor transplant”) based on their level of readiness to pursue DDKT/LDKT.

**Table 2:** **Your Path to Transplant education materials and delivery timepoints**

Timepoint	Education delivered	Time required
At baseline	▪ Computer-tailored feedback report for the individual patient based on their DDKT and LDKT readiness, decisional balance, self-efficacy, and transplant knowledge.	25 minutes
	▪ Coaching recommendations for the individual patient.	
	▪ *What You Need to Know About Kidney Transplant* Booklet introduced^a^: 19 facts about the benefits and risks of transplant and the transplant evaluation and surgery process.	
	▪ *What You Need to Know About Living Donation* Booklet introduced^a^: 15 facts about the benefits and risks of living donation and the living donation evaluation and surgery process.	
	▪ *A Community Resources Guide* introduced^a^: A guide providing referrals to community services like reduced or free childcare or transportation services, and inexpensive housing near the transplant center for patients who are facing socioeconomic barriers to transplant.	
During evaluation	▪ Computer-tailored feedback report for the individual patient based on their DDKT and LDKT readiness, decisional balance, self-efficacy, and transplant knowledge.	20 minutes
	▪ Coaching recommendations for the individual patient.	
	▪ LDKT Education, based on LDKT Readiness Stage, either:	
	○ Review and discuss *Why People Donate Their Kidneys* Brochure: Provides a list of reasons why living donors decided to donate a kidney to someone in need. Given to patients in LDKT readiness stages of Precontemplation and Contemplation.	
	○ Review and discuss *How to Find a Living Donor Brochure*: Suggests small steps potential kidney recipients can take to find potential living donors. Given to patients in LDKT readiness stages of Preparation and Action.	
	▪ *A Guide for Family & Friends Brochure*: This brochure offers small steps family members and friends can take to support the patient in completing evaluation successfully and, if interested, pursuing LDKT.	
	▪ Explore Transplant DVD: This 4-part video discusses why patients decided to pursue transplant, why living donors decide to donate, the recipient and living donor evaluation and surgery processes, the risks and benefits to transplant, and how patients lives have changed.	
4 months post-baseline	▪ Computer-tailored feedback report for the individual patient based on their DDKT and LDKT readiness, decisional balance, self-efficacy, and transplant knowledge.	15 minutes
	▪ Coaching recommendations for the individual patient.	
	▪ Additional LDKT education, as their LDKT stage of readiness changes over time.	
	▪ Additional copies of the *Community Resources Guide* to support them as their socioeconomic barriers change over time.	
8 months post-baseline	▪ Computer-tailored feedback report for the individual patient based on their DDKT and LDKT readiness, decisional balance, self-efficacy, and transplant knowledge.	15 minutes
	▪ Coaching recommendations for the individual patient.	
	▪ Additional LDKT education, as their LDKT stage of readiness changes over time.	
	▪ Additional copies of the *Community Resources Guide* to support them as their socioeconomic barriers change over time.	

^a^These materials are introduced during the education session and then mailed to the patient.

The coach will also discuss the benefits of DDKT and LDKT and provide suggestions for any concerns mentioned in the assessment (e.g., “*You are concerned that if the transplant fails, it would have been a lot of work and pain for nothing. The first thing to know is that transplants, in general, are very successful* […]”) or issues that are reducing patients’ confidence in being able to successfully pursue LDKT (e.g. *“It seems that you are not feeling very confident that you could take actions to pursue living donor transplant. Finding a living donor isn’t always easy. It can take time and can sometimes be disappointing, if family members or friends don’t volunteer or match. It’s important to be patient and creative when problems emerge, and to stay hopeful.* […]”) Finally, coaches will provide resources and strategies that may assist with overcoming socioeconomic barriers to transplant.

#### YPT coaches

The YPT Coaches, who have expertise in social work, psychology, public health, nursing and health communication, deliver the CTI-generated recommendations to patients in person and by telephone. Before conducting the program, each coach will have: 1) been assessed to be proficient in the use of the YPT computer program; 2) completed 6 hours of cultural-sensitivity training; 3) reviewed the study’s field training manual and key transplant literature; 4) participated in training about the TTM; 5) completed role play and practice coaching exercises with an Expert TTM Trainer and patients; and 6) learned about community resources available to help overcome socioeconomic barriers to transplant. A coach is trained to approach the patient in an empathetic, non-judgmental way.

#### YPT supplementary education materials

Finally, many brochures, fact sheets, and videos based on Dr. Waterman’s previous qualitative and quantitative research will be used in the YPT program [10, 33, 44, 45] (Table 2). Transplant recipients and living donors in the videos represent patients of different races, ethnicities, and socioeconomic groups. All educational materials are written for patients with low health literacy. Along with the individual feedback reports, specific combinations of these materials will be mailed in folders to review at home, based on the patient’s level of LDKT readiness at that specific time-point.

Videos will also be provided to patients in the YPT intervention condition. First, based on their level of LDKT readiness, patients will watch one of two 8-minute videos on evaluation day, either a video discussing general advantages to pursuing LDKT or a more advanced video providing practical suggestions for how to find living donors. Second, the patient will be given a one-hour *Explore Transplant* video to review at home with people who help them make important health decisions. The videos include the stories of 20 transplant recipients and living donors and discuss the questions and fears they had before getting a transplant and why they became motivated to pursue transplant. Health professionals on the video and educational fact sheets provide answers to common questions, including specifics about the evaluation, surgery and recovery processes involved with being a transplant recipient and a living donor. The benefits and risks of being a kidney recipient and a living donor are also outlined. This video is closed-captioned for the hearing impaired.

This program also invites family members and friends to learn with the patient. The patient will receive a *Guide for Family Members and Friends* that provides suggestions about how others could help the patient learn, decide what is best for them, and, potentially, be living donors themselves.

#### Community resource guide to address socioeconomic barriers to transplant

A *Community Resource Guide* will also be given to patients to provide them with referrals to services that may help them overcome socioeconomic barriers to transplant. Referral resources include access to dental services, reduced or free childcare or transportation services, and inexpensive housing near the transplant center. The coaches will refer the patients to specific resources within the guide if the patient expresses concerns about not being able to successfully pursue transplant due to these types of barriers.

### Usual Care (UC) educational components

The transplant education of kidney patients at UCLA-KPTP randomized to the UC education condition will take place predominately during evaluation at the transplant center and afterwards, by telephone, as needed (Table 3). Three to five weeks after scheduling a transplant evaluation appointment, the patient will come to UCLA-KPTP to attend a transplant educational seminar and complete a series of medical, psychological and financial tests to screen for health problems or other concerns that could affect the success of the transplant. Patients who pass all of these tests are called transplant candidates.

**Table 3:** **Usual care education materials and delivery timepoints**

Timepoint	Education delivered	Time required
During evaluation	• Power point that outlines the transplant process from evaluation to post-transplant. The second portion of the presentation focuses on financial preparation, resources for housing and transportation, and caregiver obligations post-transplant.	2 hours
	• Patient Education Handbook that contains information regarding the transplant process, the Kidney and Pancreas Transplant Program selection criteria process, transplantation and hepatitis c/HIV, the role of social services, additional information about nutrition and staying fit as well as information for potential donors and what they can expect throughout the process.	
	• Discussion with transplant coordinator, nephrologist, surgeon, financial coordinator, and social worker during evaluation.	
After evaluation	• Additional discussions with transplant coordinator, nephrologist, surgeon, financial coordinator, and social worker after evaluation.	1 hour

During evaluation, the two-hour transplant education seminar will be delivered by a social worker or nurse to a group of transplant patients and any family members or friends who are present. The transplant coordinator will deliver a PowerPoint presentation that outlines the evaluation, surgery, and recovery processes, how to financially prepare for transplant and communicate with the transplant team, possible resources for housing and transportation, and caregiver obligations post-transplant. In regards to LDKT, the coordinator discusses how to obtain a LDKT, who is eligible to be a living donor, the available paired donation programs, and what the donor can expect during evaluation and surgery. The patients receive a copy of the PowerPoint slides and a “Patient Education Manual”, that includes additional printed education regarding the transplant and living donation processes, recipient selection criteria, nutrition, and exercise. They also receive education about LDKT including two pamphlets discussing incompatible blood type and kidney transplantation and the UCLA kidney exchange program.

During their one-on-one evaluations with the transplant coordinator, social worker, nephrologist and/or surgeon, patients have the opportunity to ask additional questions. Additional educational opportunities are also available by phone after evaluation when speaking with their transplant coordinator while completing their final medical tests and tracking their progress on the waiting list.

### RCT overview

**Figure 2:**
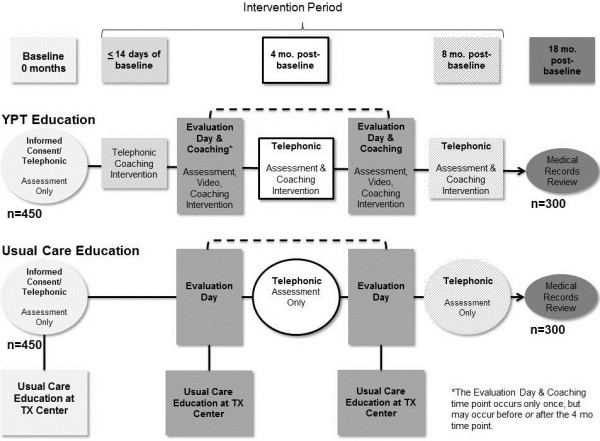
**Your Path to Transplant study design flowchart.**

In this study, we will conduct an educational intervention as Black, Hispanic, and White ESRD patients present for and undergo transplant evaluation, and take actions to pursue DDKT and LDKT. This longitudinal, parallel RCT has two treatment conditions with equal allocation, the YPT Intervention and the UC control education conditions (Figure 2). Patients in both groups will be surveyed prior to presenting at the transplant center (baseline), and at 4- and 8-months post-baseline. Patients in the intervention group will also be surveyed an additional time during their in-person transplant evaluation meeting. Completion of transplant evaluation and receipt of LDKT will be assessed for both groups using medical records 18-months post-baseline.

Patients in the YPT and UC education conditions will receive identical UCLA-KPTP education during transplant evaluation for an estimated 3 hours and will continue to learn on their own afterwards. Patients in the YPT intervention group also will receive additional sources of transplant education including individualized feedback reports, print and video education resources, and coaching at four time points: a few weeks before and during transplant evaluation, and 4 and 8 months after baseline. Patients in the intervention (YPT) condition will receive an estimated 1 hour and 15 minutes of additional formal transplant education compared to patients in the UC condition over an 8 month period.

### Patient recruitment, eligibility, randomization, and retention

The records of patients who have recently been scheduled for their evaluation day appointments will be obtained from UCLA-KPTP’s clinical database, randomized to a treatment group by a data manager, and uploaded into a patient recruitment and tracking database supported by the Research Electronic Data Capture (REDCap) software [46]. Research staff will contact patients by telephone to be invited to participate in the study. The REDCap recruitment database will include eligible patients’ names, contact information, demographic data, data on their retention in the study and progress through the survey time points, and their final clinical outcomes at the end of the trial. It will be continually updated by the study research coordinators and a data manager.

Inclusion criteria for the RCT include: 1) presentation for transplant evaluation at UCLA by calling to make an appointment; and 2) self-identification as either White, Black or Hispanic race/ethnicity. Exclusion criteria include: 1) being under age 18; 2) not being able to speak English; 3) being previously deemed ineligible for transplant at UCLA; 4) being on the wait-list for transplant at a center other than UCLA; 5) pursuing multi-organ transplant; or 6) having no consistent access to a working telephone. After being informed of the risks and benefits of the trial, patients will be asked to give verbal informed consent to participate and have their electronic medical records reviewed. This protocol has been approved by the University of California, Los Angeles’s Internal Review Board (#14-000382) and registered at ClinicalTrials.gov (#NCT02181114).

We will recruit 900 patients at baseline, 450 per treatment group. We estimate a conservative attrition rate of approximately 33%, resulting in a final sample of approximately 600 subjects, 300 in each group. To maximize participation and minimize attrition, we will provide a $10 incentive upon completion of each of the 3 or 4 time points (depending on treatment group), pay for the costs of transplant center parking and provide snacks at evaluation day, and collect alternative contact information at baseline in case of study dropout. We also have the ability to monitor patients’ progress through transplant evaluation daily using electronic medical records, which will greatly reduce the number of patients who are lost to follow-up.

### Survey timepoints

To begin the trial, we will conduct a 40-minute assessment of patients in the YPT and UC conditions to measure their demographic and clinical characteristics, any socioeconomic barriers to transplant, as well as their baseline DDKT and LDKT knowledge, decision-making, readiness, and self-efficacy. Upon beginning evaluation approximately 3–5 weeks later, patients in the YPT condition will repeat components of the survey during a 10-minute assessment. Four and 8 months later, patients in both conditions will complete two more 15-minute surveys to assess how their knowledge, decision-making, and behavior are changing over time. During the final 8-month interview, we also will ask patients questions regarding the cultural competency of their medical providers and other process measures including whether they had made an informed decision as to whether or not pursue DDKT or LDKT, and the overall helpfulness of the education they received.

For patients in the UC education condition, no additional education or coaching will occur after these surveys are administered. Patients in the UC education condition will receive a $10 gift card and a letter of thanks in the mail after each time point. Patients in the YPT condition will receive additional education and tailored coaching, as discussed next.

### YPT baseline education and coaching

Typically within 14 days post-baseline survey, patients randomized to YPT will have a separate 25-minute baseline coaching telephone call. During this call, as prompted by bulleted coaching recommendations generated by the YPT computerized program, the coach will discuss the most relevant content to support each patient in completing transplant evaluation successfully and pursuing LDKT. Immediately after the call, the coach will mail each patient a folder containing the informed consent documents, an individually-tailored feedback report generated from their answers to the baseline survey, print DDKT and LDKT educational resources, including a *Community Resource Guide*, and a $10 gift card.

### YPT evaluation day education and coaching

On evaluation day, 3 to 5 weeks after baseline, patients in the YPT intervention condition will be taken to a private area in the hospital where they will be reassessed to see how their transplant decision-making is changing. Then, the coach will provide 10-minutes of feedback based on any changes in readiness, confidence, and perceived benefits or concerns that have occurred since the initial assessment. Since the focus of this meeting is LDKT, patients also will watch a 10-minute LDKT video appropriate for their level of readiness and a discussion will be held about small steps they could take that honor their stage of readiness to further explore or pursue LDKT (20 minutes total time). At the end of the session, the coach will give the patient a second folder including an individually-tailored feedback report, print educational materials about LDKT, including an *Explore Transplant* video, educational resources to share with their family members and friends, and a $10 gift card. This meeting will take 30 minutes (20 minutes of coaching and 10 minutes for survey assessment).

### YPT 4- and 8-month education and coaching

During two additional telephone assessments at 4- and 8-months following baseline, patients will be resurveyed (15–20 minutes) to see how their decision-making and behavior is changing and, in the YPT education condition only, surveyed at 8 months about YPT’s helpfulness. Tailored coaching (15 minutes) will be given by telephone and individual feedback reports and $10 gift cards mailed following the phone call.

### YPT coaching fidelity

Throughout the intervention, the coaches will participate in weekly calls with a clinical psychologist, Dr. Mark Robbins, to discuss questions and continue to improve the tailored coaching process. Coaches administering the YPT intervention will be assessed for fidelity using a standard rubric [47]. All patient interviews will be audio recorded with the patient’s consent. For each coach, the first 10 calls will be reviewed to ensure competency, then, afterwards, a randomly selected 10% of their calls will be reviewed by two raters and checked to see if the content and dose of the intervention has been delivered consistently, per protocol. Coaches administering the intervention and coaching to the intervention group will be rated on: 1) their standardized interviewing technique; 2) the consistency of coaching to TTM principles (e.g., effective tailoring to stage of readiness); 3) effective delivery of additional trial components (e.g., addresses socioeconomic status barriers to transplant, cultural competency); and 4) effective communication skills and rapport. Coaches scoring below a minimum threshold will be required to undergo further training before continuing with the trial.

### Outcome measures

#### LDKT and DDKT readiness

Using validated measures [44, 45], patients will be asked how ready they are to get a DDKT by reporting whether they are “not considering getting a DDKT in the next six months” (Precontemplation), “considering getting a DDKT in the next six months” (Contemplation), “preparing to get a DDKT in the next 30 days” (Preparation), “undergoing evaluation to get a DDKT” (Action) or “listed and waiting to get a DDKT” (Maintenance) [44]. Patients will also be asked how ready they are to pursue LDKT and to rate whether they are “not considering taking actions in the next six months to pursue LDKT” (Precontemplation), “considering taking actions in the next six months to pursue LDKT” (Contemplation), “preparing to take actions in the next 30 days to pursue LDKT” (Preparation), or “taking actions to pursue LDKT” (Action) [45].

#### Small steps toward LDKT and DDKT

To assess their steps toward transplant, patients will be asked to respond whether they have “Already done”, “Are planning to do”, or “Don’t plan to do” a list of actions related to LDKT [45] and DDKT [44]. Examples of steps toward LDKT include “Read information/watch videos about getting a living donor transplant” and “Ask potential donors to be tested”, while examples of steps toward DDKT include “Share educational materials about deceased donation with people in your life” and “Follow-up with the transplant coordinator until transplant evaluation is complete”.

#### Decisional balance and self-efficacy

Decisional Balance measures will assess how patients weigh the relative importance of possible LDKT and DDKT positive and negative outcomes (e.g., “My living donor will feel good seeing my health improve”). Patients will be asked to rate the importance of each statement on a 5-point scale ranging from, (1) “Not important” to (5) “Extremely important” [44, 45]. The Self-Efficacy scale measures the confidence an individual has in his/her ability to pursue transplant in a wide variety of challenging situations (e.g., “If your friends and family were unsupportive of you getting a transplant”) on a scale from (1) “Not at all confident” to (5) “Completely confident” [44, 45].

#### Transplant knowledge

Patients will be asked 11 true/false and 8 multiple choice questions to determine their level of knowledge regarding basic facts, advantages, risks and outcomes of DDKT and LDKT (e.g., “Patients older than 80 years can receive transplants”; “Compared to transplants from donors who have died, how long do transplants from living donors last?”) [10]. A measure of transplant knowledge is then created by summing the number of correct answers to the questions.

#### Final transplant outcome

Finally, patients’ electronic medical records at UCLA and data from the Scientific Registry for Transplant Recipients (SRTR) will be reviewed 18 months post-baseline to create a final study outcome for each patient. At the study’s conclusion, the patient will be coded as to: 1) be waitlisted for a DDKT; 2) have received a DDKT; 3) have received a LDKT; 4) have been determined to be medically ineligible for transplant; 5) have died; or 6) have dropped out of evaluation.

### Predictors and covariates

#### Demographic, clinical and cultural factors

Many other patient characteristics will be assessed including age, sex, race/ethnicity, dialysis status (on or off dialysis; if on, hemodialysis or peritoneal), patient comorbidities (e.g., the presence of diabetes, hypertension, polycystic kidney disease) and validated measures of health-related quality of life, including the Medical Outcomes Study (MOS) SF-12v2 [48] and a patient-reported Karnofsky rating of health status [49]. To examine how other known causes for health disparities in transplant affect key outcomes we will also assess medical mistrust using the Medical Mistrust Index, a validated scale that assesses patients’ agreement with 7-items about their trust of health care organizations (E.g., “Patients have sometimes been deceived or misled by health care organizations”) [29, 50].

#### Socioeconomic transplant derailers

Multiple characteristics also are thought to be associated with having greater levels of socioeconomic stressors that may impact successful completion of transplant evaluation. These include patients’ education level, the type of health insurance they have (private, government, multiple sources, no insurance), and their employment status (full time, part time, disability, other financial assistance programs, no employment). Additional measures such as income vulnerability (e.g., “If your family lost your current income, how long could you continue to live in your current situation?”) [51], family obligations, access to transportation [52], and feelings of safety in their neighborhood will also be assessed to determine the level of vulnerability each patient faces. Lastly, patients will be asked a series of two open-ended questions to determine: 1) how personal life factors might make it difficult to get a transplant (“What are you dealing with that might get in the way of you pursuing transplant?”) and 2) what plans the patient already has in place to overcome the identified barrier (“Do you have any ideas about how you could make sure that this doesn’t stop you from pursuing transplant?”).

#### Social support and availability of living donors

Social support will be assessed by examining discordance on responses to two items measured on a 4-point Likert scale ranging from “None” to “A great deal”: “In the past 6 months, how much help or support have you received, related to your kidney disease?”; “In the past 6 months, how much help or support have you needed, related to your kidney disease?” [52]. Availability of donors will be assessed by measuring the number of close and extended family members and friends a patient has, presence of kidney disease or other comorbidities (e.g., diabetes and hypertension) in these groups, and the number of donor offers.

#### Transplant education and health literacy

Patients’ previous transplant education will be evaluated with four “Yes/No” questions that explore whether they have: 1) read brochures, 2) watched videos, 3) browsed the internet, and 4) talked to doctors or medical staff about LDKT or DDKT. Patients responding “Yes” to any of the items will then be asked how much time they have spent learning about transplant through that distinct method. Subjective health literacy and numeracy [53] will be assessed with two items scored on Likert-type scales: “How often do you have someone (like a family member, friend, hospital/clinic worker or caregiver) help you read hospital materials?”, “How confident are you filling out forms by yourself?”

#### Evaluation and process measures

At the 8 month assessment, or if patients receive a transplant or are deemed permanently ineligible for transplant before then, patients will be asked to assess the cultural competence of their healthcare providers during the evaluation process (e.g., “How often were you treated unfairly at the transplant center because of your race or ethnicity?”) on a 4-point scale ranging from “Never” to “Always”. Cultural competence will be further explored with 4 items that assess how much patients agreed that they felt trust and concern from their providers (e.g., “Did you feel you could trust them with your medical care?”; “Did you feel they really cared about you as a person?”). All cultural competence measures were adapted from the Consumer Assessment of Healthcare and Provider Systems (CAHPS) Clinician & Group Survey [54].

Patients will also be asked a series of questions about their informed decision making. The decisional conflict scale [55] was adapted to reflect three kidney disease treatment options including remaining on dialysis, getting a DDKT, and taking actions to pursue LDKT. After identifying which treatment option(s) they have chosen, patients will be asked to evaluate their decision (e.g., “I know the risks and side effects of each option”; “I am choosing without pressure from others”; “I am satisfied with my decision”) using a 5-point, Likert-type scale ranging from “Strongly Agree” to “Strongly Disagree”. To assess whether a patient has made an informed decision, patients will respond using a 4-point Likert-type scale ranging from “Completely Disagree” to “Completely Agree” to the following questions: “I have all the facts I need to make an informed decision about whether or not to pursue deceased donation” and “I have all the facts I need to make an informed decision about whether or not to pursue LDKT”.

### Data management and statistical considerations

To ensure confidentiality and security of patients, all data, including de-identified audio-recordings of patient interviews, will be stored in university-maintained, encrypted, password protected servers. Also, in accordance with NIH policy, we have established a data safety and monitoring board to monitor our trial’s progress. All study data will be captured in two electronic databases. First, registration data, patient demographics, patient tracking fields, and transplant status/medical records will be captured in a REDCap patient registration system [46]. Study personnel can check on patients’ records by examining their data entry form or through reports generated in REDCap. The records of patients who refuse to participate or are never successfully recruited to the study will be retained in the REDCap registration database and de-identified at the end of the study so that patterns in recruitment can be analyzed and reported.

Second, assessments of transplant decision-making, factors that could derail patients in their pursuit of transplant, health literacy, medical mistrust, and social support at baseline, 4 and 8 months post-baseline and relevant coaching recommendations will be captured in the YPT computerized survey and coaching program. The YPT system has been programmed using Pro-Change Behavior Systems, Inc.’s proprietary behavior change software that utilizes spreadsheets, within which statistical decision rules for generating patient feedback, assessment questions and response options, and multimedia specifications are programmed and configured. An offline Java process converts the information on these project configuration spreadsheets into Java objects, and stores the Java objects in a single serialized file. Then TTMX, a Java application, accesses the rules defined in the serialized file to give TTM-based, individualized feedback. As a participant proceeds through the program, TTMX compiles the text and graphic components of each screen based on the participant’s responses and on what have been programmed in the spreadsheets. The statistical decision rules are derived from multivariate analyses. An integrated back-end database allows for dynamic stored responses which enables dynamic tailoring. The integrated database allows for a fully-integrated user experience regardless of their chosen method of engagement (i.e., coaches, online, and others).

### Power and sample size

The sample size and power calculation for this trial was treated as a test of the proportional differences between YPT and UC patients in the Action stage of LDKT readiness at 8-months post-baseline. Based on previous work, we conservatively estimated that, at 8 months post-baseline, 10% of UC patients and 20% of patients in YPT group would reach the Action stage [10, 33]. Assuming a one-tailed alpha of 0.05, a sample size of 300 patients per group is required at 8 months post-baseline to achieve power of 0.80 (total n = 600). Assuming an 8-month post-baseline retention rate of 66%, a total sample size of 450 per education condition will need to be recruited/consented at baseline (n = 900). This sample size will provide sufficient sensitivity to detect a difference in increase in LDKT readiness (i.e., moving to Action) of about 10% between treatment (YPT) and control (UC) groups using logistic regression.

### Statistical analyses

All statistical tests will employ an intent-to-treat (ITT) approach wherein subjects will maintain their assignment to the treatment condition to which they were originally randomized regardless of whether they actually end-up receiving the planned interventions or not [56]. Multiple imputation of missing data will be used for the analyses [57]. The alpha level for all tests will be set at 0.05, and random effects models will be used to account for longitudinal measurement and linear regression employed for normally distributed outcomes, logistic regression for binomially distributed outcomes, and Poisson regression for count outcomes. The social, demographic, and other characteristics of patients who refuse to join the study or are never successfully contacted will be compared to those who do not to determine if the patient selection procedure has resulted in a biased sample. A similar analysis will be conducted to compare the patients who drop-out of the study to those who do not to determine if patient attrition has biased the sample.

To test whether, compared to the UC education condition, Black, Hispanic, and White patients receiving YPT increase in their LDKT readiness, we will first examine the proportion of individuals moving into the Action stage for readiness to accept a LDKT 8 months post-baseline between the YPT and UC conditions. For a more fine grained assessment of change in LDKT and DDKT readiness throughout the study, change by three occasions (baseline, 4 months post-baseline, and 8 months post-baseline) will be examined. To assess differences in transplant knowledge, DDKT/LDKT self-efficacy, and DDKT/LDKT decisional balance, comparisons between the YPT and UC conditions by these three occasions will be made to determine if the intervention increases transplant knowledge, DDKT/LDKT self-efficacy, and DDKT/LDKT decisional balance scales. Finally, differences in the number of small steps toward LDKT and DDKT will be examined at these three time points.

To determine whether, compared to the UC group, Black, Hispanic and White patients receiving YPT are more likely to complete transplant medical evaluation and pursue LDKT 18-months post-baseline, regression models will be fit with: 1) only the treatment condition as a predictor; 2) race added-in as a covariate; 3) an interaction term between treatment and race. To examine whether the YPT’s effectiveness in changing LDKT decision-making and behavior is different between Blacks, Hispanics and Whites, comparisons will be made of proportional forward movement in readiness at 8 months post-baseline between Black, Hispanic and White patients in the YPT condition. Also, Black, Hispanic and White patients will be compared on three occasions (baseline, 4 months post-baseline, and 8 months post-baseline) to determine if access to YPT increases transplant knowledge differently for Black, Hispanic and White patients through this follow-up period and if the intervention has different effectiveness on the increase in proportion of living donors being evaluated for LDKT and increases in the proportion of LDKTs among Black, Hispanic and White patients 18-months post-baseline.

Finally, to examine how known patient, family, and healthcare system barriers to LDKT act alone and in combination with YPT to affect Black and Hispanic patients’ decision-making and behavior around LDKT, the direct effects of key modifiable and non-modifiable variables known to affect disparities in receipt of LDKT (e.g., medical mistrust, quality of health insurance, physician recommendation to pursue LDKT) on readiness to pursue LDKT, transplant knowledge, completion of transplant evaluation, and receipt of LDKT will be adjusted for in regression models of YPT’s effect on these outcomes for Blacks and Hispanics separately. Then, interactions between YPT and these factors will be added to the models. Specifically, we will model each dependent variable first using the main effects of the barrier variables and of the YPT intervention, and then add two-way interactions between the intervention and the barrier variables. Similar models will be constructed for White patients. Based on these models, we will determine: (1) the estimated magnitude of racial differences between Black, Hispanic and White patients with and without access to the YPT for each outcome, and (2) which patient and care-related variables represent important confounding and modifying effects for explaining racial/ethnic differences in each outcome. These analyses will help elucidate whether the YPT achieves results by attenuating the impact of specific ‘causes’ of racial/ethnic disparities in LDKT.

Qualitative analyses will be performed to understand responses to key open-ended questions asked in the survey, particularly those around socioeconomic barriers to patients’ pursuit of transplant, potential solutions to barriers patients face in their transplant pursuit, and actions patients are willing to take toward LDKT and DDKT. Patients’ responses will be coded and analyzed to determine themes.

## Discussion

In every transplant center in the country, there are racial disparities in the receipt of LDKTs [58]. Active educational interventions to increase Black and Hispanic patients’ pursuit of LDKT have demonstrated some success [27, 59–62]. For example, in one dialysis center study, compared to their baseline attitudes, Blacks, younger patients, and patients who spent less time on dialysis were significantly more willing to pursue LDKT after receiving a print and video-based intervention compared to patients who did not receive this program [60]. In transplant center studies, compared with traditional clinic-based education, significantly more patients in a home-based educational intervention condition, particularly Blacks, had living donor inquiries, evaluations, and LDKTs [61, 63, 64]. Further, two important trials are underway that will develop and assess the effectiveness of: 1) culturally sensitive education to improve kidney disease patients’ early consideration (pre-ESRD diagnosis) of LDKT with and without the assistance of a social worker [65]; 2) group- and individual-based LDKT education at patients’ homes and in transplant centers [66]. Less interventions aimed at Hispanic patients have been conducted, though a culturally sensitive transplant evaluation process for Hispanic patients providing linguistic support in Spanish and transplant education that focuses specifically on concerns among Hispanic patients was successful in increasing Hispanic patients’ knowledge of and positive attitudes about living donor transplant [27].

While these trials have, or promise to, contribute important evidence about how best to reduce racial disparities in LDKT, a common limitation among them is that they are not tailored to patients’ individual readiness, self-efficacy, or confidence to pursue LDKT. The proposed YPT RCT is novel because it assesses the effectiveness of an individually-tailored health education intervention and personalized coaching system to reduce racial disparities in LDKT. The findings of this trial will foster greater understanding of how YPT specifically affects White, Black and Hispanic patients’ LDKT decision-making, knowledge, and behavior.

Another novel feature of the YPT trial is its ability to examine the impact of socioeconomic factors that may derail White, Black and Hispanic patients’ pursuit of LDKT. Past studies have demonstrated that patients of lower socioeconomic status are less likely to successfully complete transplant evaluation [14, 67]. However, these studies have compared the relative effects of few socioeconomic factors simultaneously [10, 11, 68], focused exclusively on factors that are difficult to modify (e.g., type of health insurance or household income) [11, 68], or employed measurement at the neighborhood or census block level [14, 68, 69]. In YPT, after assessing key, specific barriers and providing support services to address them, we will be able to pinpoint the factors most likely to interfere with evaluation completion and successful LDKT.

In conclusion, through this trial, we will have developed a computerized *Your Path to Transplant* tailored computer intervention that could be utilized by providers serving ESRD patients in 250 transplant centers nationwide to tailor LDKT education by race/ethnicity and based on the needs of the individual patient. With additional testing in other clinical settings and other patient populations, this YPT program could be used in other healthcare settings including in community nephrologists’ offices, in dialysis centers, and even on the internet with patients and their families learning at home. Only then can all ESRD patients be assured the opportunity to learn about and pursue the treatment option most beneficial to them – LDKT.

## Abbreviations

ESRD: End-stage renal disease
LDKT: Living donor kidney transplantation
YPT: Your Path to Transplant computer-tailored intervention
UC: Usual care education (control condition)
DDKT: Deceased donor kidney transplant
ET: Explore Transplant
TTM: Transtheoretical model of behavioral change
CTI: Computer-tailored intervention
UCLA: University of California, Los Angeles
UCLA-KPTP: University of California, Los Angeles Kidney-Pancreas Transplant Program
RCT: Randomized controlled trial
SRTR: Scientific Registry of Transplant Recipients
MOS SF-12: Medical Outcomes Study Short Form 12
CAHPS: Consumer Assessment of Healthcare and Provider Systems
NIH: National Institutes of Health
IRB: Internal review board
REDCap: Research Electronic Data Capture
ITT: Intent-to-treat
OPTN: Organ Procurement and Transplantation Network.
